# Mental Health and Childhood Adversities: A Longitudinal Study in Kabul, Afghanistan

**DOI:** 10.1016/j.jaac.2010.12.001

**Published:** 2011-04

**Authors:** Catherine Panter-Brick, Anna Goodman, Wietse Tol, Mark Eggerman

**Affiliations:** a Yale University; b London School of Hygiene & Tropical Medicine

**Keywords:** family risk, conflict, resilience, violence, posttraumatic stress disorder

## Abstract

**Objective:**

To identify prospective predictors of mental health in Kabul, Afghanistan.

**Method:**

Using stratified random-sampling in schools, mental health and life events for 11-to 16-year-old students and their caregivers were assessed. In 2007, 1 year after baseline, the retention rate was 64% (n = 115 boys, 119 girls, 234 adults) with no evidence of selection bias. Self- and caregiver-rated child mental health (Strengths and Difficulties Questionnaire), depressive (Depression Self-Rating Scale), and posttraumatic stress (Child Revised Impact of Events Scale) symptoms and caregiver mental health (Self-Report Questionnaire) were assessed. Lifetime trauma and past-year traumatic, stressful, and protective experiences were assessed.

**Results:**

With the exception of posttraumatic stress, one-year trajectories for all mental health outcomes showed significant improvement (*p* < .001). Family violence had a striking impact on the Strengths and Difficulties Questionnaire data, raising caregiver-rated scores by 3.14 points (confidence interval [CI] 2.21–4.08) or half a standard deviation, and self-rated scores by 1.26 points (CI 0.50–2.03); past-year traumatic beatings independently raised self-rated scores by 1.85 points (CI 0.03–3.66). A major family conflict raised depression scores by 2.75 points (CI 0.89–4.61), two thirds of a standard deviation, whereas improved family life had protective effects. Posttraumatic stress symptom scores, however, were solely contingent on lifetime trauma, with more than three events raising scores by 5.38 points (CI 1.76–9.00).

**Conclusions:**

Family violence predicted changes in mental health problems other than posttraumatic stress symptoms in a cohort that showed resilience to substantial socioeconomic and war-related stressors. The importance of prospectively identifying impacts of specific types of childhood adversities on mental health outcomes is highlighted to strengthen evidence on key modifiable factors for intervention in war-affected populations.

Afghanistan is a challenging setting in which to undertake child/adolescent mental health research. One of the five poorest countries in the world,^1^ its public health profile bears witness to a noxious combination of ongoing conflict and chronic poverty. Access to health care has markedly improved^2^ since the 2001 ousting of the Taliban regime, as have educational opportunities for children,^3,4^ but pronounced inequalities remain.^5^ Two large-scale surveys have documented, for adults, traumatic experiences, loss of social functioning, and a spectrum of poor mental health outcomes.^6,7^ Recent studies^8-11^ have focused attention on Afghan youth, in response to global concern for child/adolescent mental health in war zones.^12-14^ Such work has drawn attention to the mental health impact of daily stressors and societal violence, namely threats to psychological well-being that are not solely consequent on war.^15^ All work to date, however, has been cross-sectional, unable to discern the prospective impact of different kinds of adverse exposures.

In conflict areas, mental health research has primarily focused on war-related trauma and posttraumatic stress disorder rather than a broader set of predictor and outcome variables, and individuals rather than families as units of analysis and intervention.^8,16,17^ Few longitudinal “naturalistic” studies of youth in community settings are available,^18^ with noteworthy exceptions in Mozambique,^19^ Iraq,^20,21^ Gaza,^22^ and Sierra Leone,^23^ and fewer still encompass family-level research. One key debate^16,24^ focuses on the relative importance of exposure to different kinds of militarized, domestic, and structural violence, namely whether mental health outcomes are primarily driven by war-related trauma, family-level violence, and/or structural barriers taking the form of institutional, social, and economic stressors. Most existing surveys, however, have focused on single childhood adversities predicting single disorders, rather than clusters of adversities and changes over the life course.^25^ Even in low- and middle-income countries unaffected by war, few prospective studies of children and adolescents have teased out the relative impact of area-level, family-level, and individual-level predictors of poor health.^26^ Thus, when it comes to the predictors of child/adolescent mental health, much less is known about the impact of neighborhood, social class, family conflict, and parental depression than about individual-level predictors such as age, sex, and war-trauma exposure.

In 2006, we conducted a school-based survey to establish baseline mental health data for 11- to 16-year-olds and adult caregivers (n = 1,011 child–adult pairs) in three regions of the country,^10^ including 364 children and 364 caregivers in the capital Kabul. We also collected extensive qualitative data on psychosocial suffering, resilience, and everyday stressors in face-to-face interviews with the 1,011 children and 1,011 adult respondents.^11^ One year later, we recontacted Kabuli participants to reappraise risk factors and assess intervening-year events. This article reports on the sample with repeated measures at baseline (T1) and follow-up (T2), focusing on youth but using caregiver data where relevant to characterize family environments. We examined changes in mental health over time, including individual and contextual risk/protective factors, using a wider set of mental health indicators than traditionally studied for war-affected children. Specifically, we hypothesized that intervening-year events (relating to individual, family, and neighborhood circumstances) and baseline risk factors (such as lifetime trauma and gender) would predict T1 to T2 trajectories. To inform existing debates, we empirically tested the prospective impact of ongoing individual and social stressors and the sustained impact of lifetime trauma exposure.

## Method

### Research Design

**FIGURE 1:**
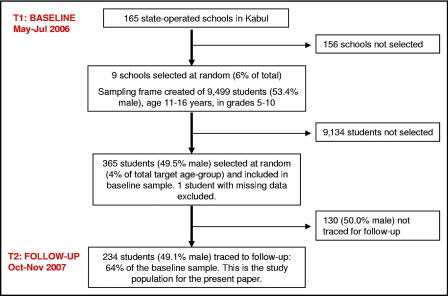
Sample selection: two-stage stratified random sampling in schools.

In Afghanistan, schools provide the best setting to interview a community-based sample of male/female children/caregivers. Nationally, 64% of 7- to 14 year-olds (48% girls, 77% boys) enrolled in school in 2004 through 2005.^3^ There are formidable cultural barriers to interviewing male/female participants in other settings, such as mosques or homes, given security concerns and restricted opportunity for interview privacy. Our baseline survey (T1: May through July 2006) adopted a stratified random-sampling design across several regions. The follow-up (T2: October through November 2007) was conducted only in Kabul, due to heightened insecurity and logistic constraints, with the same field team (three male, three female interviewers, a professional translator, and a bilingual project manager). At T1, we achieved balanced gender and geographic coverage of 6% of listed schools and 4% of target-age students (Figure 1). We contacted government-operated schools, with probability sampling proportional to size and additional stratification by single-sex/coeducational schools and city zones.^10^ We compiled age-specific class lists in selected schools and randomly sampled 11- to 16-year-olds, excluding siblings. At T2, we recontacted the same schools and reinterviewed 64.3% of students and primary caregivers; adults who assumed day-to-day childcare responsibility were, in 61.5% of cases, the same person at baseline and follow-up.

The protocol was approved by international and local ethics committees, including the Ministry of Education in Afghanistan. Written informed consent was obtained from school directors, oral consent from children, caregivers, and teachers, and procedures for potential referral of participants with physical/emotional problems were specified.^27^ All participants agreed to the T2 interview, given good rapport built at T1, a small gift, and a free health examination. Given an absence of systematic record-keeping at schools, it was not possible to trace students who had left; their families were lost to follow-up.

### Mental Health Indicators

We developed two-language versions (Dari/Pashtu) of several standardized rating scales recommended for epidemiologic research in schools and/or conflict settings, including Muslim communities in Pakistan, Bangladesh, Bosnia, and Gaza.^10^ We selected brief, locally applicable questionnaires with demonstrated psychometric properties to assess mental health problems including emotional/behavioral/social difficulties, depressive, and posttraumatic stress symptoms and closely adhered to procedures for preparing such instruments for transcultural research.^28,29^ Translations and backtranslations were reviewed for content validity and cultural relevance during 2 years of extensive preparatory work. This included focus groups, panel review, and two pilot surveys to assess the content validity and psychometric properties of instruments in Afghanistan in samples of 320 child–adult pairs and a 7-day test-retest of reliability in a Kabul sample of 20 respondents. Our reviewing panel consisted of Afghan trilingual fieldworkers and academics with interdisciplinary expertise, including one Afghan clinical psychologist, one British expert in child/adolescent psychiatry, and one American clinical psychologist with field experience in Afghanistan. We did not attempt to establish criterion validity of the rating scales, because this would have required long-term time investments on the part of mental health professionals who are but a handful in Afghanistan, and operate within an extremely incapacitated health care system.^30^ Our research was to identify prospective predictors of mental health using dimensional outcomes.

For children, we implemented the Strengths and Difficulties Questionnaire (SDQ), the Birleson Depression Self-Rating Scale (DSRS), and the Child Revised Impact of Events Scale (CRIES) at both time points. The SDQ is an internationally well-validated 25-item questionnaire providing balanced coverage of behavioral, emotional, and social problems for multi-informant completion.^31-33^ Four subscales assess emotional, behavioral, hyperkinetic, and peer problems, yielding a total difficulty score (range 0–40) for the previous 6 months. A fifth subscale taps prosocial strengths. Supplementary questions measure the impact (none/minor/definite/severe) of a child's difficulties in terms of distress and interference in everyday life. The SDQ permits explicit comparison of self-rated and parent-rated scores about the same child: multirespondent scores are usually discrepant but significantly correlated, and the SDQ performs well compared with other outcome indicators reviewed in the literature.^32^ Single-informant SDQ ratings have been validated in Bangladesh, Pakistan, Yemen, and Gaza.^10^ Notably, the total difficulty score is a genuinely dimensional measurement of child mental health across its full range.^33^ The Dari/Pashtu versions we developed in Afghanistan were copyrighted to www.sdqinfo.org. They demonstrated good internal reliability (Cronbach α = 0.66 for self-rated, α = 0.77 for caregiver-rated SDQ total difficulty scores, n = 364) and test-retest reliability (Spearman Brown *r* = 0.57, *p* = .009, n = 20).

The DSRS (18 items, 3-point scale) and the CRIES (13 items, 4-point scale) are widely used in disaster and conflict settings to assess, respectively, depressive symptoms and posttraumatic stress symptoms. CRIES was implemented only for children reporting trauma exposure, because intrusion/avoidance items measuring levels of distress consistent with posttraumatic stress disorder are tied to specific traumatic experiences. Dari/Pashtu versions showed good internal reliability (DSRS, α = 0.692; CRIES, α = 0.820) and 7-day test-retest reliability (*r* = 0.756 and *r* = 0.783, respectively, *p* < .0001).

We implemented the Self-Report Questionnaire (SRQ-20) with all caregivers. This is a simple and effective measurement of the burden of common mental health problems (20 items, yes/no answers), with good internal reliability (α = 0.83) in our study. We previously established excellent overlap with the Afghan Symptom Checklist, an instrument developed in Kabul to measure psychological distress with culturally specific terminology.^10^

### Traumatic, Stressful, and Protective Experiences

We assessed lifetime traumatic events and past-year experiences, conducting detailed evaluations of a variety of psychometric properties to follow recommendations for transcultural epidemiology in humanitarian settings.^29^

To develop a locally relevant Traumatic Events Checklist, we reviewed the child-focused 17-item Gaza Traumatic Event Checklist^34^ and an adapted version of the Harvard Trauma Questionnaire^35^ implemented with adults in Afghanistan.^6^ Our expert panel selected items most pertinent to Afghan children's experiences. In common with the Gaza instrument,^22^ the checklist content is specific to traumatic events experienced in the wake of war and displacement in Afghanistan, rather than encompassing events common to war in other settings. We included 20 yes/no events, differentiating direct experience from witnessing or hearing reports of prespecified events, plus one item for “any other” traumatic experience. These assessed lifetime trauma pertaining to serious injuries due to knife/gunshot/explosion, severe physical beatings, forced displacement, home expulsion, enforced family separation, direct exposure to bombardments/rocket explosions, a family member killed/wounded as a result of war, and danger to one's life. Afghan panel members insisted that a question on rape be removed, because it was likely to be offensive and elicit poor-quality data in the context of securing interviews with children and caregivers; it was deemed unethical to proceed with this question. Extensive piloting showed that only one item (on torture) needed clarification. To contextualize checklist yes/no responses, we asked respondents to describe each event, identify their most distressing traumatic event, and how long ago it happened. Such descriptions served to categorize each trauma report in terms of lifetime versus past-year exposures of family-level, community-level, and political violence. Checklist item test-retest reliabilities ranged from κ = 0.643 to κ = 1.000 (*p* = .002 to *p* < .0001).

We assessed past-year stressors and protective factors with a separate checklist. Stressors included 15 items regarding threats to health, family events, loss of friendships, financial circumstances, domestic and community conflict, and “any other” event. Protective factors included 12 items regarding improved health, friendships/neighborhood relationships, family and home circumstances, and area living/security conditions (plus coping ability, school/work situations, perceived neighborhood trust, and other area-level conditions; data not tabulated). Such items were identified as culturally relevant from extensive content analyses of 1,011 child and 1,011 adult T1 interviews and subsequent panel review. As in other studies that developed culturally grounded survey instruments,^15,36^ we incorporated items phrased in local terminology. For example, to prompt reports on domestic violence, we asked “has anyone in your family been violent or bad-tempered toward other family members?”—the expression “bad-tempered” (Dari: *bad-khulqi*, lit. ill-natured, amoral) is a culturally acceptable way to signal the presence of abusive and violent domestic conflict. To tap protective factors, we asked about family “harmony and unity” (Dari: *ittifaq* and *wahdat*)—terms describing the quality of within-household relationships. We randomly selected item starting points across respondent interviews using 3-point show cards to illustrate ratings on current status (bad/so-so/good), intervening-year changes (worse/same/better), and burden (not at all/only a little/quite a lot/a great deal).

Full demographic and socioeconomic data were collected from caregivers. These included household composition, displacement history, parent educational/occupational data, child education/work activities, number of wage earners, type of household material possessions, and purchasing ability. In terms of economic vulnerability, caregivers self-evaluated their household as food insecure (very poor), unable to buy items such as clothing (poor), able to afford most commodities (average), or to cover all their needs (better off).

### Statistical Analyses

We present self-rated SDQ, caregiver-rated SDQ, self-rated DSRS, and self-rated CRIES scores as outcome measurements for child mental health. We used caregiver SRQ-20 as a predictor variable for child outcomes and compared child/caregiver outcomes to examine consistency of risk factors for Afghan families. We adjusted for clustering by school (using STATA 10.1, STATA Corporation, College Station, TX) to produce robust standard errors and tested potential effect modification (interaction with sex).

To rule out selection bias, we compared participants lost with retained to follow-up for sex, age, ethnicity, years of schooling, scholastic performance, household wealth, demographic composition, displacement history, father/mother educational and occupational status, and child/caregiver mental health. Sensitivity analyses testing alternate socioeconomic indicators, linear/categorical data for trauma events, child- or caregiver-only reports yielded similar findings. To assess cohort-level changes, we restricted analyses to respondents with T1 and T2 data (n = 234 children, n = 234 adults for all outcomes, except n = 79 for CRIES after trauma). For children, we examined whether SDQ cohort-level changes were merely consistent with age-related changes observed cross-sectionally in the larger baseline dataset (n = 1,011). For adults, we undertook sensitivity analyses restricted to the same person interviewed at T1 and T2 (n = 144).

To identify prospective predictors of mental health, we tested the impact of past-year traumatic, stressful, and protective experiences, adjusting for a priori baseline factors (T1 mental health, sex, age, lifetime trauma, socioeconomic position). Because child/adult respondents might differentially report items such as family-level violence and area-level security, we used sensitivity analyses to demonstrate that results were similar whether based on child-only, caregiver-only, or “any” child/caregiver reports, and tabulated analyses for “any” child/caregiver data. Analyses were conducted in two steps. First, to assess the salience of potentially stressful (n = 15) and protective (n = 12) experiences, we ran separate multivariate regressions on each item; given multiple testing, we focused on results significant at *p* ≤ .01. We ran separate regressions on past-year traumatic exposure to family-level violence (two items, experiencing or witnessing severe beatings), community-level violence (neighborhood stabbings/beatings/brutalities), and political violence (witnessing people killed/injured by Taliban or suicide bombings). Second, we built multivariate models to test the relative contribution of predictor variables, including past-year variables with demonstrated statistical significance in the first analytical step, and correcting for the same five baseline predictors.

## Results

The follow-up sample consisted of 234 children (mean ± standard deviation [SD] = 13.5 ± 1.51 years old) and 234 caregivers (35.7 ± 10.9 years). Caregivers included 43 fathers and 101 mothers who were primarily responsible for boys and girls, respectively, and 90 other close relatives. In 38.5% of cases, illness or work meant that close relatives other than T1 informants had assumed primary childcare responsibilities at T2. Children/caregivers lost to follow-up did not differ in their baseline characteristics from those retained in the study (Table 1); no evidence of participation bias was found regarding demographic, educational, socioeconomic, or baseline mental health characteristics. One in four students (26.4%) worked in paid/unpaid jobs (at market stalls, in apprenticeships, or carpet-weaving) before or after school. Most families were food insecure (41.5%) or poor (18.0%); most (82.1%) had been displaced at least once during the child's lifetime.

As expected, SDQ and DSRS outcomes were significantly and moderately intercorrelated (n = 234, *r* = 0.39, *p* < .001, for self- and caregiver-rated SDQ; *r* = 0.54, *p* < .001, for DSRS and self-rated SDQ). For the subsample of 79 children with lifetime trauma exposure, posttraumatic stress disorder symptoms correlated significantly and moderately with other outcomes (*r* = 0.34, *p* < .001, for CRIES and DSRS; *r* = 0.42, *p* < .001, for CRIES and self-rated SDQ). In addition, there were bivariate associations between caregiver- and self-rated SDQ scores (*r* = 0.39, *p* < .001) at both time points. Adult SRQ-20 was associated with child self-rated SDQ (*r* = 0.25, *p* < .0001) and CRIES (*r* = 0.21, *p* = .01) but not with DSRS (*r* = 0.05, *p* = .42).

**FIGURE 2:**
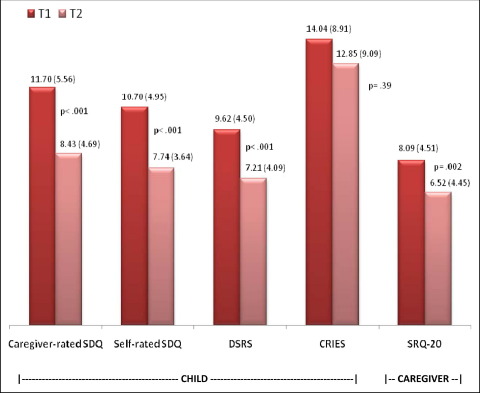
Changes in mental health from baseline (T1) to follow-up (T2). Note: Mean (SD) scores, with *p* values for T1-T2 changes (two-tailed *t* tests, corrected for clustering by school). CRIES = Child Revised Impact of Events Scale; DSRS = Depression Self-Rating Scale; SDQ = Strengths and Difficulties Questionnaire (total difficulties); SRQ-20 = Self-Report Questionnaire.

From T1 to T2, we observed a significant improvement for all mental health outcomes except posttraumatic stress symptoms (Figure 2). SDQ scores decreased by 3 points whether self-rated (10.70 to 7.74, *p* < .001) or caregiver rated (11.70 to 8.43, *p* < .001); impact scores for reported difficulties decreased (*p* = .01 for self- and caregiver ratings), whereas scores for prosocial functioning increased (*p* = .007 self-rated, *p* < .01 caregiver rated). Changes were consistent across all subscales (emotional, conduct, hyperkinetic, peer problems, prosocial functioning, plus supplementary impact questions), significant for male and female subjects, and consistent across all ages. Likewise, depressive symptoms decreased (9.62 to 7.21, *p* < .001). Adult mental health also improved, with SRQ-20 decreasing by 1.6 points (*p* = .002); the magnitude of change was similar for male (5.86 to 4.43) and female (10.18 to 8.47) subjects, with the 2-point gender gap significant (*p* < .001) at both time points, whether or not analyses were restricted to the same informant at T1 and T2. In contrast, CRIES scores decreased by only 1 point, a nonsignificant trend for this subsample.

In terms of past-year risk factors, four family-level exposures had notable impact on child outcomes (Table 2). For SDQ, the strongest predictor was family-level violence/*bad-khulqi*: self-rated SDQ changed by 1.60 points (*p* = .003) and caregiver-rated SDQ by 3.19 points (*p* < .001). Major family conflicts increased DSRS by 3.21 points (*p* = .006) and serious family illnesses by 2.02 points (*p* = .009). Past-year trauma reports of family violence (witnessing/experiencing severe physical beatings at home) also predicted self-rated SDQ changes (2.66 points, *p* = .006). For CRIES, no variable reached significance (*p* < .01). It is notable that caregivers were similarly affected by family violence/*bad-khulqi* and serious family illness (increasing SRQ-20 by 2.69 points, *p* < .001, and 1.63 points, *p* = .005, respectively). Results were consistent across child-only or caregiver-only data, despite some child–adult inconsistency in reporting life events. For example, the occurrence of family violence/*bad-khulqi* was reported in 109 families (47%) by at least one informant, but in only 37 families (16%) by both informants, producing an adult–child κ value of 0.30. The level of agreement was similar when restricting the analysis to events found stressful (i.e., incorporating burden), with an adult–child κ value of 0.27. However, this variable had a consistent prospective impact on child and adult mental health trajectories, whether reported by children or caregivers, for exposure or stressful burden. Moreover, 27 families reported severe physical beatings at home to be traumatic, rather than a merely stressful, experience; this trauma also predicted child-rated SDQ outcomes. In contrast, socioeconomic stressors, community-level violence, and political violence showed nil or weak associations.

In terms of past-year changes in protective factors, three items were noteworthy (Table 3). Changes in “family life” was associated with child SDQ (*p* = .009) and DSRS (*p* = .02) and caregiver SRQ-20 (*p* = .03), with better family life predicting better outcomes (*r* = −1.52, *r* = −2.13, and *r* = −1.54, respectively) as evidenced by the negative values of regression coefficients. Two other variables, household financial circumstances and neighborhood living conditions, affected DSRS. No other item had detectable impact on data variation.

Table 4 presents final multivariate analyses for identifying prospective predictors of child mental health, correcting for five baseline variables (sex, lifetime trauma, socioeconomic position, child and caregiver mental health); we included salient past-year trauma exposure (severe physical beatings at home), stressful risk factors (serious family illness, family violence/*bad-khulqi*, major family conflict), and protective factors (better family life, household financial circumstances, neighborhood living conditions). We assessed three self-rated child mental outcomes (SDQ, DSRS, CRIES), adjusting for baseline. For SDQ, three family items exerted significant, independent, and prospective effects: scores changed by 1.85 points (confidence interval [CI] 0.03–3.66) with traumatic beatings, 1.26 points (CI 0.50–2.03) with stressful violence/*bad-khulqi*, and −1.48 points (CI −2.26 to −0.69) with reports of better family life, changes of one half to one third of SD (0.51, 0.35, and 0.41, respectively, where outcome SD = 3.64). For DSRS, a major family conflict increased scores by 2.75 points (CI 0.89–4.61), or two thirds of a SD (0.67, where SD = 4.09), independently from a serious family illness. In contrast, no intervening-year event predicted CRIES variation. We also assessed two caregiver-rated outcomes (child SDQ, adult SRQ-20). For children, family violence/*bad-khulqi* increased SDQ by 3.14 points (CI 2.21–4.08), or two thirds of a SD (0.67, where SD = 4.69). For adults (R^2^ = 0.48, not tabulated), it increased SRQ-20 by 2.15 points (CI 1.11–3.20), or one half the SD (0.48, with SD = 4.45), with better family life having independent a protective effect (−1.63 points; CI −2.71 to −0.54).

Baseline characteristics also predicted T1-T2 trajectories. With lifetime exposure to at least three trauma events, CRIES increased by 5.38 points (CI 1.76–9.00). Lifetime trauma also predicted changes in caregiver-rated SDQ: the association with time depth of the most distressing event was nonsignificant (adjusted *r* = −0.13, CI −0.34 to 0.08), but in the expected direction of decreasing scores with increasing years since exposure. Being female predicted a higher self-rated SDQ by 1.29 points (CI 0.22–2.37). Baseline child mental health scores, but not baseline caregiver scores, significantly predicted child SDQ and DSRS past-year trajectory: once we adjusted for individual baseline scores, caregiver mental health did not prospectively and independently predict child mental health.

## Discussion

To overcome some of the limitations of current knowledge on child mental health in war-affected settings, we followed a representative sample of schoolchildren (n = 234) and caregivers (n = 234) in Kabul, retaining 64.3% of the initial student sample 1 year after baseline. We provide evidence that family violence is a prospective predictor of poor mental health outcomes, even where cohort-level mental health outcomes improve over time, except for posttraumatic stress symptoms, where lifetime trauma exposure trumps all other risk factors.

With respect to family-level predictors, we observed an interesting pattern of risk and protective factors. Traumatic domestic beatings, stressful family violence/*bad-khulqi*, and stressful family conflict had notable prospective effects in fully adjusted multivariate models (Table 4). One measurement of domestic violence incorporated a Dari phrase identified through baseline qualitative analyses of open-ended interviews with 1,011 children and 1,011 adults^10,11^ to be a culturally relevant way of reporting abuse in Afghanistan. This cultural sensitivity may explain why this measurement picked up the most instances of family violence: 109 families reported past-year occurrence of family violence/*bad-khulqi*, which in 84 cases was “quite” or “very” burdensome; in contrast, just 27 reported enduring or witnessing physical beatings severe enough to be reported as a traumatic experience. Greater cultural sensitivity and greater statistical power (due to higher frequency) may also explain why the stressful “violence/*bad-khulqi*” measurement showed consistent effects across SDQ outcomes for sensitivity analyses on child-only, adult-only, or “any” reports, whereas the “traumatic physical beatings” measurement was associated with self-rated SDQ but not with caregiver-rated SDQ. Notably, family violence affected both child and caregiver well-being, whereas better home life had consistent protective effects. The SDQ results are clinically relevant: each 1-point increase in parent-reported and child-reported SDQ corresponds to an increased probability of clinician-assigned mental disorder, a relation that holds across the full dimensional range of scores.^33^

These findings underscore conclusions from World Health Organization global mental health surveys regarding the salience of “maladaptive family functioning” as a type of childhood adversity, one that predicts long-term (adult) psychopathology.^25^ In their accounts, Afghan respondents differentiated between adversities that were acceptable, stressful, or frankly traumatic.^11^ Domestic beatings, for instance, are a normative form of “disciplinary violence,” whereby corporal punishment castigates poor school results, mistakes at work (e.g., carpet weaving before/after school), or imparts discipline (as in the statement of a 15-year-old boy: “if the father is away from home, the uncle beats younger members of the family and the women, […] this is normal for an uncle to beat his brother's wife”). “Everyday violence,” however, is attributed to psychological ill-health—a father beating family members “with a cable and sticks because he had a troubled mind,” or a mother beating children due to frustration with her own circumstances. Family conflict often worsens at the point of adolescence, when boys come under increasing pressure to work full time, and girls to marry. Indeed, violence can poison “family harmony” to the point of attempted suicides: some adolescents reported having been rushed to hospital after ingesting rat poison, and female caregivers having wanted to throw themselves off the roof “because of all the beatings.”^11^

With respect to past-year exposures, we did not detect associations between traumatic community/political violence and SDQ/DSRS scores. This is noteworthy, despite the small samples, because participants knew of one recent suicide bombing that had resulted in the deaths of children on a school trip, had witnessed suicide bomb attacks at bus stops/police stations, or seen the aftermath of such attacks; to give a striking example, one student reported seeing blood and “a burqa thrown all the way up an electricity pylon” just 4 hours after the event. Thus collective violence might be less salient than proximate family environments for prospective impact on mental health outcomes such as child SDQ/DSRS or adult SRQ-20. This is in contrast to trauma exposure predicting posttraumatic stress: for two in five students (n = 79, 38%) reporting trauma exposure, cumulative trauma events predicted CRIES at both time points, with no other risk/protective factors and no cohort-level improvement noted.

This does not mean that political violence is unimportant in understanding common mental health problems in this context. On the contrary, domestic violence is often a response to structural and collective violence: Afghan respondents^11^ clearly articulated linkages between abusive interpersonal relationships and the enormous pressure of socioeconomic stressors and political insecurity. Our longitudinal study confirms cross-sectional work in two Kabul schools^8^ that highlighted the importance of domestic over war-related events in terms of lifetime and recent exposures, but this is in context of systemic linkages between interpersonal, structural, and collective violence, as highlighted in Sri Lanka^8,37^ and Palestine.^38,39^

Why cohort-level outcomes, except posttraumatic stress, improved over time (Figure 2) remains unknown. In the absence of a concerted mental health intervention, SDQ improved by 3 points, DSRS by 2 points, and SRQ-20 by 1.6 points, and changes were consistent across male and female subjects. SDQ scores do improve in the age range under consideration,^11^ but 3-point changes are more than expected.^40^ We carefully evaluated response bias,^27^ running a systematic postfieldwork evaluation to appraise participant expectations and potential exaggeration of T1 responses to trigger assistance. We found a remarkable consistency across SDQ subscales, a good indication that the screening instrument functioned as expected. Systematic response biases cannot be discounted but are likely not the sole explanation for cohort-level changes. We found no evidence of selection bias, although 37% of students were lost to follow-up. Likely reasons for sample attrition are residence changes—8 in 10 families had already experienced forced displacement (Table 1)—and economic/cultural obligations to curtail education.

We cannot link cohort-level changes to improved security conditions in Kabul; in 2006 through 2007, there were sharp increases in the number and scale of suicide attacks^41^ and an increased threat from the Taliban who targeted the capital.^42^ There was, however, urban reconstruction in terms of large-scale road-building and electricity provision. Two thirds of respondents (62%) stated that the security situation remained the same in their home area, whereas 6% saw deterioration over the past year; half the sample reported better living conditions and better social interactions in the neighbourhood (52% and 56% respectively, Table 3). Our data may indicate a measurement of resilience^43^ to protracted armed conflict, as evidenced by improved mental health despite pervasive structural stressors and constant violence. “Natural remission” has been reported in a few observational^20,22^ and most intervention^44-46^ longitudinal studies of war-affected youth, even in the absence of clear sociopolitical changes. Such work has noted the importance of school integration as a protective factor for children, in addition to the impact of hopelessness and despair on adult capacities to provide good parenting and support.^20,47^ In Afghanistan, the ability of families to maintain psychosocial and material resources and particularly to remain geographically stable, economically robust, and socially supportive enough to keep near-adolescent boys and girls in school for yet another year may capture an important facet of resilience. In our follow-up, 234 families (from 364 at baseline) managed to keep their children in their current school; of these, over the intervening year, 45 moved home, 16 were threatened with eviction, 51 lost a wage earner, and 178 incurred a substantial debt (Table 2). Our qualitative data^11^ show that resilience was expressed as life “feeding on hope,” because children focused on school as the gateway to socioeconomic advancement to alleviate economic stressors and maintain family unity. In this sense, the follow-up sample consisted of families able to anchor their children in school, a significant expression of hope and resilience in a high-risk environment.

Study limitations are those common to other work in conflict settings: small samples, reports subject to recall and subjective biases, reliance on screening instruments rather than clinical diagnoses, and generalizability of findings (limited in the present study to school-attending children). In addition, caregiver data are limited in that respondents—designated primary caregivers in the child's household—were the same person at T1 and T2 in only 61.5% of cases. We therefore could not fully adjust for caregiver mental health at baseline. Our study, however, has notable strengths. We interviewed a representative, randomly selected sample of male/female students and caregivers, an achievement for Afghanistan. In particular, we recruited female caregivers for private face-to-face interviews in a culture where access to randomly selected female informants is usually denied (for some mothers, customarily secluded at home, our survey was their first-ever opportunity to even visit their children's school). We were unable to include families who deemed state-provided education as socially unacceptable or even unaffordable, yet our sample is highly comparable to national data available for Afghanistan (28.9% school-aged children working outside the home^48^ and 44% of households in the poorest, i.e., food-insecure, category^49^). We also achieved a 64% sample retention rate, with no evidence of selection biases. Moreover, we established in a separate Kabul study^50^ that self-reports of family-level stressors are associated with physiological measurements of stress (blood pressure and cell-mediated immune responses). We integrated culturally grounded data in screening instruments and assessed measurement reliability and consistency across multi-informant reports. As such, we provide original family-level research to contribute to a growing body of work on children/adolescents exposed to violence; in low- and middle-income countries, this has been identified as a top-ranking priority for mental health research.^51^

The literature has identified an urgent need for policies and practices that support families to meet the mental health needs of children and adolescents,^52-54^ taking the stance that families are the most important resource for fostering mentally healthy individuals.^55^ In conflict settings, this lesson needs strong reiteration: family-level violence is a consistent predictor of changes in mental health trajectories, even in a context of ongoing exposure to war-related violence. Our prospective work strengthens the call for targeted interventions that address mental health difficulties consequent on domestic, not just war-related, violence,^37^ and the linkages between individual and collective exposures to pervasive violence.^24^ Because intervention requires detection,^25^ it also highlights the value of using simple yet effective measurements to track mental health in individual children,^56^ to identify key modifiable factors that will sharpen the focus of structural, community-based, and individual-level interventions. This is especially relevant in Afghanistan, where the Ministry of Public Health included mental health as one of seven priorities within its Basic Package of Health Services, to bridge the huge gap between existing service provision and community-level needs through a decentralization of mental health services.^57^ One landmark study has argued that violence toward children as an expression of punishment and control is accepted, but not condoned, in Afghanistan.^58^ There is still little public debate on the issue of domestic violence, but mounting evidence that developing effective public health, education, and family-strengthening interventions directed at decreasing family-level conflict requires urgent action. In conclusion, our work has policy implications consistent with reviews examining the evidence base for a wider range of childhood adversities^25^ and mental health outcomes, for differential pathways linking specific exposures to specific outcomes,^59^ and for effective child- and family-focused mental health interventions in resource-poor and humanitarian settings.^13,60^ Specialized mental health interventions need to serve children reporting posttraumatic stress, for whom symptoms may persist over time, but family-based and structural interventions need to address ongoing family violence, a type of childhood adversity that has sizeable prospective impact on common mental health problems.

## Figures and Tables

**TABLE 1:** Sample Characteristics at Baseline, by Follow-up Status (n = 364 children)

	Lost to Follow-up	Retained for Follow-up	
	(n = 130)	(n = 234)	*p*^a^
Sex (%)			
child			
male	50.0	49.1	.92
female	50.0	50.9	
caregiver			
male	40.8	48.3	.42
female	59.2	51.7	
Mean age (y)			
child	13.7	13.5	.41
caregiver	37.2	35.7	.36
Socioeconomic position (%)			
household			
food insecure	48.5	41.5	.57
poor	18.5	18.0	
average	16.2	23.1	
better off	16.9	17.5	
Lifetime traumatic events (%)			
child			
0 event	26.2	43.6	.08
1–2 events	40.8	35.0	
≥3 events	33.1	21.4	
caregiver			
0 event	1.5	5.1	.06
1–2 events	23.1	27.8	
≥3 events	75.4	67.1	
Mean child mental health scores			
self-rated SDQ	11.5	10.7	.37
caregiver-rated SDQ	12.8	11.7	.13
DSRS	10.4	9.6	.20
CRIES	15.1	12.8	.19
Mean caregiver mental health scores			
SRQ-20	9.13	8.09	.23

Note: CRIES = Child Revised Impact of Events Scale (for children with trauma exposure); DSRS = Depression Self-Rating Scale; SDQ = Strengths and Difficulties Questionnaire (total difficulties); SRQ-20 = Self-Report Questionnaire.

a χ^2^ tests and two-tailed t tests.

**TABLE 2:** Past-Year Risk Factors (Stressful and Trauma Events) and Prospective Impact on Child Mental Health

Indicators	Events	Total Events, N	Events Found Stressful, n (%)	Impact of Stressful Events on T1–T2 Changes (Adjusted Regression Coefficients)			
Self-Rated SDQ	Caregiver-Rated SDQ	DSRS	CRIES
(N = 234)	(n = 234)	(n = 234)	(n = 234)	(n = 234)	(n = 79)
Stressors^a^							
Health	death in family	40	39 (98%)	0.49	0.16	1.14⁎	4.14⁎
	family member seriously ill	184	165 (90%)	0.68⁎	1.17⁎	2.02⁎⁎	3.57⁎
Family events	family member moved out	43	23 (53%)	0.10	−0.35	0.35	few cases
	family member just married	67	11 (16%)	−0.06	−0.04	1.28	few cases
	birth in household	80	1 (1%)	few cases	few cases	few cases	few cases
	family moved home	45	34 (76%)	1.18⁎	1.32	0.89	4.71
Friendships	been separated/lost contact with close friend	37	27 (73%)	0.66	−0.11	2.02⁎	4.82
Financial circumstances	family member lost salaried job	51	32 (63%)	−0.19	1.06	0.18	0.02
	family member incurred any substantial debt	178	163 (92%)	0.04	0.66	1.03	2.47
	family paid for large social gathering or celebration	109	41 (38%)	1.22	1.21⁎	1.64⁎	1.31
	family threatened with eviction from home	16	14 (88%)	0.71	1.77	0.59	few cases
	home overcrowded	126	47 (37%)	0.10	0.18	0.19	3.07
Conflict and violence	family member violent or *bad-khulqi* at home	109	84 (77%)	1.60⁎⁎⁎	3.19⁎⁎⁎	1.42	5.06⁎
	family involved in major conflict or dispute	21	15 (71%)	1.51	1.99	3.21⁎⁎	few cases
	family member attacked, beaten or robbed	6	5 (83%)	few cases	few cases	few cases	few cases
Trauma^b^	family-level violence (severe physical beatings)	27	27 (100%)^a^	2.66⁎⁎	1.01	1.31	8.40⁎
	community-level violence (stabbings/beatings/brutalities)	19	19 (100%)^a^	−0.62	−1.03	−1.62	−0.55
	political violence (killings/beatings/suicide bombings)	14	14 (100%)^a^	1.09	0.31	0.20	few cases

Note: Multivariate regression analyses (each item in turn) adjusting for baseline mental health score, sex, age, socioeconomic position, and lifetime trauma exposure. Events reported by fewer than 10 people were not used in analyses. Results were similar for male/female subjects, with no interaction by sex. CRIES = Child Revised Impact of Events Scale (for children with trauma exposure); DSRS = Depression Self-Rating Scale; SDQ = Strengths and Difficulties Questionnaire (total difficulties); T1 = baseline survey; T2 = follow-up.

a Checklist of past-year stressful events, defined as “quite” or “very” burdensome.

b Traumatic Events Checklist, past-year exposure.

⁎ *p* ≤ .05;

⁎⁎ *p* ≤ .01;

⁎⁎⁎ *p* ≤ .001.

**TABLE 3:** Past-Year Protective Factors and Prospective Impact on Child Mental Health

Indicators	Compared With 1 y Ago, How Do You Rate . . .	n (%)	Impact on T1–T2 Changes (Adjusted Regression Coefficients)			
Self-Rated SDQ	Caregiver-Rated SDQ	DSRS	CRIES
(n = 234)	(n = 234)	(n = 234)	(n = 79)
Health	your physical health?					
	worse	36 (15%)	2.24	0.66	0.11	3.00
	same	118 (50%)	0⁎	0	0	0
	better	80 (34%)	−0.05	−0.19	−0.55	0.30
Friends and neighbors	your friendships with other people?					
worse	4 (2%)	few cases	few cases	few cases	few cases
	same	78 (33%)	0	0	0	0
	better	152 (65%)	−0.22	−0.22	−0.41	−0.59
	your interactions with people in the neighborhood?					
	worse	6 (3%)	few cases	few cases	few cases	few cases
	same	97 (41%)	0	0	0	0
	better	131 (56%)	0.46	0.93	1.19	0.92
Family and home life	your family life at home?					
worse	21 (9%)	0.94	0.40	−1.35	few cases
	same	154 (66%)	0⁎⁎	0	0⁎	0
	better	59 (25%)	−1.52	−0.80	−2.13	−5.71
	your family's harmony/unity (Dari: *ittifaq/whahdat*)					
	worse	13 (6%)	1.98	−0.15	−0.28	few cases
	same	93 (40%)	0	0	0	0
	better	128 (55%)	−0.44	−0.23	−0.20	−2.59
	your household's financial circumstances?					
	worse	52 (22%)	−0.30	−0.33	−1.69	2.78
	same	130 (56%)	0	0	0⁎⁎	0
	better	52 (22%)	−0.84	−0.88	−2.48	−1.65
Area living conditions	living conditions in neighborhood?					
worse	3 (1%)	few cases	few cases	few cases	few cases
	same	110 (47%)	0⁎	0	0⁎⁎	0⁎
	better	121 (52%)	0.73	0.91	1.65	2.69
	security situation in area you live in?					
	worse	15 (6%)	0.79	0.55	1.67	few cases
	same	144 (62%)	0	0	0	
	better	74 (32%)	−0.59	−0.64	−1.70	−1.03

Note: Multiple regression analyses (each item in turn) adjusting for baseline mental health score, sex, age, socioeconomic position, and lifetime trauma exposure. The *p* values are from tests for heterogeneity; substantive findings were unchanged using tests for linear trend. “Worse” categories based on fewer than 10 people were merged with “same” categories for analysis. Results similar for male/female subjects, with no interaction by sex. CRIES = Child Revised Impact of Events Scale (for children with trauma exposure); DSRS = Depression Self-Rating Scale; SDQ = Strengths and Difficulties Questionnaire (total difficulties); T1 = baseline survey; T2 = follow-up.

⁎ *p* ≤ .05;

⁎⁎ *p* ≤ .01.

**TABLE 4:** Risk/Protective Factors and Prospective Impact on Child Mental Health

Model	n	Self-Rated SDQ R^2^ = 0.28 (n = 234)	Caregiver-Rated SDQ R^2^ = 0.40 (n = 234)	DSRS R^2^ = 0.21 (n = 234)	n	CRIES R^2^ = 0.27 (n = 79)
Baseline						
Sex						
male	115	0⁎	0	0	41	0
female	119	1.29 (0.22 to 2.37)	0.06 (−1.47 to 1.58)	1.05 (−0.28 to 2.37)	38	2.72 (−1.61 to 7.06)
Lifetime traumatic events						
0	102	0	0⁎⁎	0	—	not applicable
1–2	85	0.44 (−0.59 to 1.47)	1.16 (0.60 to 1.72)	0.78 (−0.57 to 2.13)	41	0⁎⁎
≥3	50	−0.47 (−1.75 to 0.81)	−0.11 (−1.22 to 1.00)	0.04 (−1.40 to 1.47)	38	5.38 (1.76 to 9.00)
Socioeconomic position						
food insecure	97	0	0	0	38	0
poor	42	−0.11 (−2.01 to 1.79)	0.56 (−1.12 to 2.23)	−0.53 (−2.08 to 1.03)	17	0.93 (−3.95 to 5.80)
average	54	−0.32 (−1.52 to 0.89)	0.52 (−0.37 to 1.40)	−0.31 (−1.38 to 0.76)	13	0.52 (−7.84 to 8.89)
better off	41	0.14 (−1.19 to 1.46)	0.51 (−1.28 to 2.29)	0.33 (−0.77 to 1.43)	11	−3.85 (−10.57 to 2.87)
Child baseline score^a^						
change per point	234	0.20 (0.09 to 0.30)⁎⁎	0.36 (0.27 to 0.46)⁎⁎⁎	0.22 (0.02 to 0.42)⁎	79	−0.03 (−0.33 to 0.27)
Caregiver baseline SRQ-20						
change per point	234	0.00 (−0.12 to 0.12)	0.04 (−0.12 to 0.20)	0.07 (−0.05 to 0.19)	79	−0.03 (−0.33 to 0.27)
Past year^b^						
Severe physical beatings at home (trauma)						
no	207	0⁎	—	—	66	—
yes	27	1.85 (0.03 to 3.66)			13	
Family member violent/*bad-khulqi* at home (stressor)						
no	150	0⁎⁎	0⁎⁎⁎	—	52	—
yes	84	1.26 (0.50 to 2.03)	3.14 (2.21 to 4.08)		27	
Family involved in a major conflict (stressor)						
no	164	—	—	0⁎⁎	57	—
yes	70			2.75 (0.89 to 4.61)	22	
Family member seriously ill (stressor)						
no	69	—	—	0⁎	26	
yes	165			1.58 (0.16 to 3.01)	53	
Better family life at home (protective factor)						
no	146	0⁎⁎	—	—	53	—
yes	88	−1.48 (−2.26 to −0.69)			26	
Better household financial circumstances						
no	136	—	—	0	53	—
yes	98			−0.99 (−2.09 to 0.10)	26	
Better living conditions in neighborhood						
no	84	—	—	0	31	—
yes	150			1.05 (−0.32 to 2.42)	48	

Note: Multivariate model yielding adjusted regression coefficients (95% confidence interval) for tabulated variables plus respondent age. CRIES = Child Revised Impact of Events Scale (for children with trauma exposure); DSRS = Depression Self-Rating Scale; SDQ = Strengths and Difficulties Questionnaire (total difficulties).

a Outcome-specific score.

b Any report by child or caregiver.

⁎ *p* ≤ .05;

⁎⁎ *p* ≤ .01;

⁎⁎⁎ *p* ≤ .001.
